# A New Species of *Macellicephaloides* Uschakov, 1955 (Annelida, Polynoidae) from Cold Seeps in the South China Sea: Insights into the Taxonomy and Phylogeny of *Macellicephaloides* and Related Genera

**DOI:** 10.3390/cimb47110897

**Published:** 2025-10-29

**Authors:** Jie Li, Linlin Zhang, Mingxiao Wang, Xuwen Wu

**Affiliations:** 1 Laboratory of Experimental Marine Biology, Institute of Oceanology, Chinese Academy of Sciences, Qingdao 266000, China; lijie@qdio.ac.cn (J.L.); linlinzhang@qdio.ac.cn (L.Z.); 2 Laboratory for Marine Biology and Biotechnology, Qingdao Marine Science and Technology Center, Qingdao 266000, China; 3 Laboratory of Marine Organism Taxonomy and Phylogeny, Qingdao Key Laboratory of Marine Biodiversity and Conservation, Institute of Oceanology, Chinese Academy of Sciences, Qingdao 266071, China; 4 CAS Key Laboratory of Marine Ecology and Environmental Sciences, Center of Deep Sea Research, Institute of Oceanology, Chinese Academy of Sciences, Qingdao 266071, China; wangminxiao@qdio.ac.cn; 5 College of Marine Science, University of Chinese Academy of Sciences, Beijing 101408, China

**Keywords:** polychaetes, systematics, morphology, deep-sea, chemosynthetic environment

## Abstract

*Macellicephaloides* Uschakov, 1955 (Annelida: Polynoidae) is a genus of deep-sea polychaetes characterized by a specialized pharynx bearing two pairs of jaws (with the dorsal pair fused) and three pairs of lateral papillae, the middle pair of which is greatly elongated, and remarkable adaptability to diverse deep-sea habitats. Most species in this genus inhabit abyssal depths (>7200 m), with high diversity in western Pacific trenches, while a few occur in relatively shallow habitats such as deep-sea seamounts and hydrothermal vents. This paper presents a new species, *Macellicephaloides lingshuiensis* sp. nov., found in deep-sea cold seeps in the South China Sea, representing the shallowest distribution record for the genus to date and the first record from cold seep environments. The classification and phylogeny of *Macellicephaloides* and related genera have long been the subject of debate. A previous study suggested that *Macellicephaloides* is nested within the *Macellicephala* clade, but our analyses—based on 13 mitochondrial protein-coding genes, *12S*, *16S*, *18S*, *28S* rRNA, and *ITS1-ITS2* sequences—tentatively indicate that these two genera form independent evolutionary clades. Additionally, our phylogeny indicates a close evolutionary relationship between deep-sea *Macellicephaloides* and cave-dwelling polynoids (e.g., *Gesiella*), highlighting ecological connections between deep-sea and cave habitats. These conclusions are supported by morphological comparisons and genetic distance analyses. Although the subfamily Macellicephalinae is recovered as a monophyletic group, intergeneric phylogenetic relationships within it remain unresolved, highlighting the need for additional data from more species and genera. We amend the generic diagnosis of *Macellicephaloides* and provide an identification key to all valid species in the genus. This study clarifies the taxonomy and phylogeny of *Macellicephaloides* and related taxa, emphasizing the importance of continued sampling in understudied deep-sea habitats to enhance our understanding of their biodiversity.

## 1. Introduction

Deep-sea polynoids exhibit remarkable morphological diversity, which has led to their classification into numerous subfamilies and genera [1]. Among these, *Macellicephaloides*, akin to *Macellicephala*, is a genus characterized by the absence of lateral antennae and exhibiting fewer body segments and elytra. However, it is distinguished by its specialized pharyngeal morphology, including a unique configuration of papillae and jaws: specifically, a fused dorsal jaw paired with an unfused ventral jaw pair, two pairs of subequal dorsal papillae, three pairs of lateral papillae (with the middle lateral pair being elongated and cirriform), and a pair of collar-like ventrolateral folds.

Due to its unique morphological traits, the classification of *Macellicephaloides* has undergone multiple revisions. The genus was established by Uschakov [2] and initially assigned to the subfamily Polynoinae. It was subsequently transferred to the subfamily Lepidonotinae by Hartman [3] and then to the subfamily Macellicephalinae by Hartmann-Schröder [4]. Pettibone [5] further revised the classification by establishing the subfamily Macellicephaloidinae to accommodate this genus. However, based on integrated molecular and morphological data, Bonifácio & Menot [6] synonymized Macellicephaloidinae along with nine other subfamilies within Macellicephalinae, thereby reassigning *Macellicephaloides* to Macellicephalinae [7].

Phylogenetic relationships involving *Macellicephaloides* remain the subject of considerable debate. In the morphological phylogenies of Polynoidae [6], *Macellicephaloides* constitutes a well-supported monophyletic group, but its relationship with the closely related *Macellicephala* remains unresolved. In contrast, a recent molecular study by Hiley et al. [8] contradicted this morphological evidence, suggesting that *Macellicephaloides* is nested within the *Macellicephala* clade. This assertion has been challenged by the striking morphological disparities observed between the two genera. This incongruity underscores the necessity for re-evaluating the phylogenetic position of *Macellicephaloides* to elucidate its evolutionary history within Polynoidae.

To date, nine valid species of *Macellicephaloides* have been described. The majority of these species inhabit abyssal zones exceeding 7200 m, while a few species occur in relatively shallow deep-sea habitats such as seamounts and hydrothermal vents. Levenstein [9] hypothesized that the genus is likely to be widespread across Pacific abyssal regions, with the western Pacific potentially serving as its center of origin. However, current knowledge of its species diversity remains incomplete, emphasizing the need for further exploration.

During the examination of polynoid specimens collected from the ‘Lingshui’ cold seep in the South China Sea, an undescribed species of *Macellicephaloides* was identified. This species is herein named *Macellicephaloides lingshuiensis* sp. nov. In order to resolve its phylogenetic placement, we conducted molecular phylogenetic analyses using 13 mitochondrial protein-coding genes (*PCGs*), alongside *12S*, *16S*, *18S*, *28S* rRNA, and *ITS1-ITS2* sequences. The aim of this study is threefold: firstly, to provide a detailed description of the new species; secondly, to review the taxonomy, distribution, and diversity of *Macellicephaloides*; and thirdly, to investigate morphological differences and phylogenetic relationships between *Macellicephaloides* and related genera (e.g., *Macellicephala*, *Gesiella*).

## 2. Materials and Methods

### 2.1. Specimen Collection and Morphological Examination

Specimens were collected from a deep-sea cold seep in the South China Sea of the tropical Western Pacific, during the 326th dive of the ROV *FaXian* (Discovery) deployed from the R/V *KeXue* (Science) (111°03′16″ E, 17°37′20″ N; depth: 1759 m). After collection, the specimens were preserved in an 80% ethanol which is the standard practice for long-term preservation of voucher specimens in zoology, as it effectively preserves both morphological integrity and DNA for molecular studies. Subsequently, the specimens were deposited at the Marine Biological Museum of the Chinese Academy of Sciences (MBMCAS), housed at the Institute of Oceanology, Chinese Academy of Sciences (IOCAS).

Complete specimens were imaged using a Sony Alpha 7R IV mirrorless camera (Sony Corporation, Tokyo, Japan) paired with a Laowa FF 100 mm f/2.8 2x macro lens (ChangGeng Optics Technology Co., Ltd. Hefei, China). The morphology of the head, pygidium, parapodia, and chaetae was observed using a Zeiss Discovery V20 stereomicroscope (Zeiss Corporation, Oberkochen, Germany) and imaged using an Axiocam 512 colour camera (Zeiss Corporation, Oberkochen, Germany) mounted on the stereomicroscope. Images from different focal planes were combined using Helicon Focus 7 software v8.3.8. For detailed observation and photography of chaetae, selected parapodia were detached from the specimens, rinsed in absolute ethanol, dehydrated through a graded ethanol series, coated with gold, examined and photographed using a Zeiss GeminiSEM 500 scanning electron microscope (Zeiss Corporation, Oberkochen, Germany).

### 2.2. DNA Extraction, Library Preparation, and Genome Skimming Sequencing

Genomic DNA was extracted from specimens of *Macellicephaloides lingshuiensis* sp. nov. using the *Magnetic Animal Tissue Genomic DNA Kit* (Tiangen Biotech, Beijing, China), following the manufacturer’s protocol. DNA quality was assessed using a NanoDrop spectrophotometer (ThermoFisher Scientific Corporation, Waltham, MA, USA) with the OD_260_/_280_ ratio ranging from 1.8 to 2.0, and a minimum quantity of 3 μg in 50 μL elution buffer. Next Generation Sequencing (NGS) libraries were prepared with an average insert size of 300 bp and whole-genome sequencing was performed on an Illumina NovaSeq 6000 platform (2 × 150 bp paired-end, PE150) by Novogene Co., Ltd. (Beijing, China). Approximately 10 Gb of raw sequencing data were generated, ensuring sufficient coverage for downstream mitogenome assembly and analysis.

### 2.3. Mitogenome Assembly and Annotation

Raw sequencing reads were quality-checked using FastQC v0.12.1 [10]. Adapters of raw reads were trimmed using Trimmomatic v0.39 [11] (default parameters). The mitochondrial genome was de novo assembled using MitoZ v3.6 [12] (“All” mode) with a multi-kmer strategy (--kmers_megahit 59 79 99 119 141). For mitochondrial genome annotation, we first used the annotated results of MitoZ, followed by validation using MITOS2 WebServer (https://usegalaxy.eu/root?tool_id=mitos2, 18 August 2025) [13] under the invertebrate mitochondrial genetic code (NCBI transl_table = 5) and the RefSeq63 Metazoan reference database (default parameters), (https://usegalaxy.eu/root?tool_id=mitos2, 18 August 2025). Finally, thirteen protein-coding genes (*PCGs*) and two ribosomal RNAs (*12S* and *16S* rRNAs) were fully annotated, and these sequences were further verified via online BLAST [14] (https://blast.ncbi.nlm.nih.gov/Blast.cgi, 18 August 2025) searches to ensure annotation accuracy.

### 2.4. Extracting Nuclear Genes from NGS Data

To incorporate nuclear markers into phylogenetic analyses, nuclear 18S, 28S, and ITS1-ITS2 genes were extracted from the genome-skimming reads of the newly sequenced sample and additional species (including Acholoe squamosa, Eunoe nodosa, Harmothoe impar, Pettitbonesia furcosetosa, and Trypanobia cryptica) for which these nuclear genes were not previously reported but NGS data are available in the NCBI database [15] (https://www.ncbi.nlm.nih.gov/sra/, 18 August 2025). Reference sequences from closely related polynoids were retrieved from NCBI for each gene. Reads were assembled into contigs using Megahit v1.2.9 [16], followed by local Nucleotide-Nucleotide BLAST v2.17.0 [14] search against the reference database to identify target genes. Finally, identified sequences were extracted using SeqKit2 v2.2.0 [17].

### 2.5. Species Delimitation and Phylogenetic Analyses

Two distinct sequence datasets were compiled for genetic distance estimation and phylogenetic reconstruction. The first dataset comprised eight *COI* sequences, including those of four *Macellicephala* species, *Macellicephaloides alvini*, *Macellicephaloides lingshuiensis* sp. nov., Macellicephalinae sp., and *Gesiella jameensis*. The second dataset for comprehensive phylogenetic analysis was composed of sequences from 39 selected species (Table 1), including 13 *PCGs*, *12S*, *16S*, *18S*, and *28S* rRNA genes, and *ITS1-ITS2* regions. The ingroup included 32 polynoid species, while the outgroup was represented by seven species from other Aphroditiformia families, including Acoetidae, Aphroditidae, Eulepethidae, and Iphionidae, as well as a more distantly related Syllidae species to ensure robust phylogenetic rooting (Table 1).

All sequences (newly generated and those derived from GenBank; see Table 1) were aligned using MAFFT v7.526 [18]. The PCG sequences were frame-adjusted via 5′-terminal truncation to eliminate premature stop codons while performing MAFFT in PhyloSuite v1.2.3. Alignments for *PCGs* were carried out in “codon mode”, while for rRNA and *ITS* genes, the “normal mode” was used with the “auto” strategy (automatically selects the most appropriate algorithm based on sequence characteristics, with default parameters). The initial PCG alignments were then refined using MACSE v2.03 [19] in “codon mode” (-prog refineAlignment) to optimize the alignment with respect to the amino acid translation and ensure all indels were in-frame. Subsequently, the *MACSE*-refined alignments were manually inspected in PhyloSuite v1.2.3 [20] to identify and remove any large, ambiguous gap regions. RNA and *ITS* genes’ *MAFFT* alignments and *PCGs’* refined alignments were trimmed using GBlocks v0.91b [21] to remove hypervariable regions. Parameters for trimming included: a minimum block length of 5, allowed gap positions set to “with half”, “nucleotide mode” for rRNA genes and “codon mode” for *PCGs*. Processed alignments were concatenated into an 18-gene supermatrix (13 *PCGs* + *12S* + *16S* + *18S* + *28S* + *ITS1-ITS2*) using PhyloSuite v1.2.3.

The *COI* dataset was extracted from the refined alignments and used to calculate interspecific genetic distances using the Bootstrap variance estimation methods under the Maximum Composite Likelihood (MCL) model of MEGA12 v12.0.11 [22]. The subsequent phylogenetic workflow was implemented through PhyloSuite using both maximum likelihood (ML) and Bayesian inference (BI) methods. The concatenated dataset was partitioned by genes, with the 13 PCGs treated as 3-sites codon. Optimal substitution models and partition schemes for *IQ-TREE* [23] and MrBayes [24] were determined using ModelFinder (in IQ-TREE2 v2.2.0) under the AICc (Corrected Akaike Information Criterion).

For ML analysis, it was implemented in IQ-TREE2 v2.2.0, with 20,000 standard bootstrap replicates for nodal support estimation. For BI analysis, it was conducted in MrBayes 3.2.7a, involving 50 independent runs (each with four Markov chains), 50 million generations (sampling every 1000 generations), and with a 25% burn-in (12,500 trees were discarded). Convergence was confirmed by effective sample size (ESS) values exceeding 200, as assessed in Tracer v1.7.1 [25].

Final phylogenetic trees were visualized and annotated using *iTOL* [26] (https://itol.embl.de/, 18 August 2025). Nodal support values were indicated by posterior probabilities (BI/PP) and ML bootstrap values (ML/BP). Nodes were considered well-supported if they met either of the following criteria: BP ≥ 70% or PP ≥ 0.95 [27,28].

**Table 1:** Sampling information of the species and corresponding accession numbers used in this study.

Family	Subfamily	Organism	Mitogenome	18S	28S	ITS	References
Acoetidae	-	*Panthalis oerstedi*	KY753832	KY753846	KY753846	KY753846	Zhang et al. [29]
Aphroditidae	-	*Laetmonice producta*	KY753833	KY753853	KY753853	KY753853	Zhang et al. [29]
Eulepethidae	-	*Eulepethus nanhaiensis*	KY753834	KY753850	KY753850	KY753850	Zhang et al. [29]
Iphionidae	-	*Iphione* sp. YZ-2018	KY753835	KY753852	KY753852	KY753852	Zhang et al. [29]
Polynoidae	Polynoinae	*Acholoe squamosa*	OX439053	PRJEB60118	PRJEB60118	PRJEB60118	Adkins et al. [30]
Polynoidae	Admetellinae	*Admetella levensteini*	PQ221480	PQ211133	PQ211133	PQ211133	Wu et al. [1]
Polynoidae	Admetellinae	*Admetella multiseta*	PQ221478	PQ211131	PQ211131	PQ211131	Wu et al. [1]
Polynoidae	Admetellinae	*Admetella nanhaiensis*	PQ221483	PQ211136	PQ211136	PQ211136	Wu et al. [1]
Polynoidae	Admetellinae	*Admetella undulata*	PQ221482	PQ211135	PQ211135	PQ211135	Wu et al. [1]
Polynoidae	Arctonoinae	*Arctonoe vittata*	MZ131647	-	-	-	Park et al. [31]
Polynoidae	Macellicephalinae	*Branchinotogluma hessleri*	MW783686	OM007982	OM105845	-	Hiley et al. [8]
Polynoidae	Macellicephalinae	*Branchiplicatus cupreus*	MW783699	OM007993	OM105856	-	Hiley et al. [8]
Polynoidae	Macellicephalinae	*Branchipolynoe segonzaci*	OP648300	-	-	-	Hiley et al. [8]
Polynoidae	Eulagiscinae	*Eulagisca gigantea*	OP648301	OM008007	OM105872	-	Hiley et al. [8]
Polynoidae	Polynoinae	*Eunoe nodosa*	NC_060302	SRR14996616	SRR14996616	SRR14996616	Kim et al. [32]
Polynoidae	Macellicephalinae	*Gesiella jameensis*	MW794260	MW794263	MW794263	MW794263	Gonzalez et al. [33]
Polynoidae	Lepidonotinae	*Halosydna* sp. YZ-2018	KY753830	KY753845	KY753845	KY753845	Zhang et al. [29]
Polynoidae	Polynoinae	*Harmothoe impar*	OX381722	PRJEB60118	PRJEB60118	PRJEB60118	Adkins et al. [34]
Polynoidae	Lepidonotinae	*Hyperhalosydna striata*	MW620990	-	-	-	Kim et al. [32]
Polynoidae	Macellicephalinae	*Lepidonotopodium fimbriatum*	MW783701	OM007994	OM105858	-	Hiley et al. [8]
Polynoidae	Lepidonotinae	*Lepidonotus clava*	OW387151	-	-	-	Darbyshire et al. [35]
Polynoidae	Lepidonotinae	*Lepidonotus* sp. YZ-2018	KY753831	KY753851	KY753851	KY753851	Zhang et al. [29]
Polynoidae	Macellicephalinae	*Levensteiniella kincaidi*	MW783703	OM007995	OM105860	-	Hiley et al. [8]
Polynoidae	Macellicephalinae	*Macellicephala* sp. 1 AH-2024	OP648303	OM008006	OM105871	-	Hiley et al. [8]
Polynoidae	Macellicephalinae	*Macellicephala* sp. 2 AH-2024	OP648304	OM008001	OM105866	-	Hiley et al. [8]
Polynoidae	Macellicephalinae	*Macellicephala* sp. 3 AH-2024	OP648305	OM008004	OM105869	-	Hiley et al. [8]
Polynoidae	Macellicephalinae	*Macellicephala* sp. 4 AH-2024	OP648306	OM008005	OM105870	-	Hiley et al. [8]
Polynoidae	Macellicephalinae	Macellicephalinae sp.	MW816923	OM007998	OM105863	-	Hiley et al. [8]
Polynoidae	Macellicephalinae	*Macellicephaloides alvini*	OP648307	OP651045	OP651057	-	Hiley et al. [8]
Polynoidae	Macellicephalinae	*Macellicephaloides lingshuiensis* sp. nov.	PX118544	PX132557	PX132557	PX132557	This Study
Polynoidae	Macellicephalinae	*Mamiwata piscesae*	MW783702	MW654532	OM105859	-	Hiley et al. [8]
Polynoidae	Polynoinae	*Melaenis* sp. YZ-2018	KY753829	KY753849	KY753849	KY753849	Zhang et al. [29]
Polynoidae	Macellicephalinae	*Peinaleopolynoe orphanae*	MW783706	OM007999	OM105864	-	Hiley et al. [8]
Polynoidae	Macellicephalinae	*Pelagomacellicephala iliffei*	MW794261	MW794264	MW794264	MW794264	Gonzalez et al. [33]
Polynoidae	Polynoinae	*Pettitbonesia furcosetosa*	SRR16188832	SRR16188832	SRR16188832	SRR16188832	Filée et al. [36]
Polynoidae	Polynoinae	*Polyeunoa laevis*	MN057924	KU738176	KU738176	KU738176	Bogantes et al. [37]
Sigalionidae	Sigalioninae	*Euthalenessa festiva*	KY753837	KY753839	KY753839	KY753839	Zhang et al. [29]
Sigalionidae	Pisioninae	*Pisione* sp. YZ-2018	KY753836	KY753844	KY`753844	KY753844	Zhang et al. [29]
Syllidae	Syllinae	*Trypanobia cryptica*	KR534503	SRR2006109	SRR2006109	SRR2006109	Aguado et al. [38]

## 3. Results

### 3.1. Genetic Distance and Phylogenetic Analyses

A total of eight complete *COI* gene sequences, comprising 1534 nucleotide sites, were retrieved from the mitochondrial genomes of *Macellicephaloides* and closely related species. The interspecific genetic distances (*p-distance*) among the selected species ranged from 0.042 to 0.226 (see Table S1). Genetic distances within the genus *Macellicephala* ranged from 0.048 to 0.132. *Macellicephala* sp. 4 AH 2024 is particularly distinctive, showing relatively large genetic distances (0.119–0.132) from other *Macellicephala* species, while distances between other *Macellicephala* species range from 0.048 to 0.059. In comparison, intraspecific genetic distances within the genus *Macellicephaloides* can be as high as 0.199 (e.g., between *M. alvini* and *M. lingshuiensis* sp. nov.). It is noteworthy that *Macellicephaloides alvini* and *Macellicephala* sp. 3 AH 2024 show the lowest genetic distance (0.042), a pattern replicated between *Macellicephaloides alvini* and other *Macellicephala* species (0.050–0.134). Additionally, *Gesiella jameensis* and an unclassified Macellicephalinae species (Macellicephalinae sp.), also show a relatively close genetic distance (0.103).

**Figure 1:**
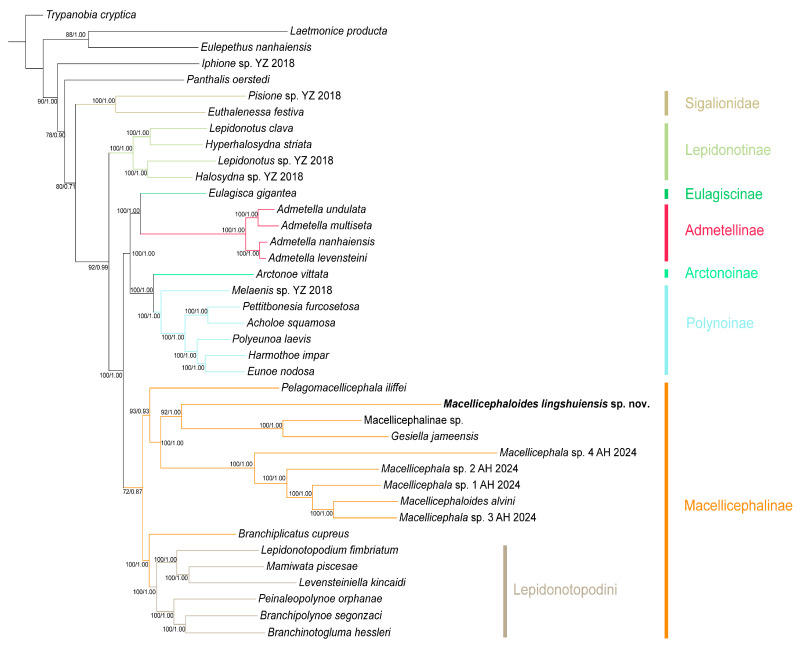
Phylogenetic tree of *Macellicephaloides* and related genera constructed by the Bayesian inference (BI) and Maximum likelihood (ML) methods using concatenated sequences (13 mitochondrial protein-coding genes (*PCGs*), *12S* rRNA, *16S* rRNA, *18S* rRNA, *28S* rRNA, and *ITS1-ITS2* genes). Numbers at the nodes represent ML bootstrap scores (left)/BI posterior probability (right). The colors of branches and strips represent different subfamilies or tribe.

Both the maximum likelihood (ML) and Bayesian inference (BI) analyses produced congruent tree topologies, with posterior probabilities (PP) and bootstrap percentages (BP) indicated at each node (Figure 1). Most nodes in the phylogenetic reconstructions received high statistical support, indicating high resolution and reliability of the inferred topology. The resulting phylogenetic tree topology revealed six polynoid subfamilies, with some forming well-supported clades, and a well-supported outgroup clade from the family Sigalionidae.

Macellicephalinae (BP/PP = 72/0.87): As the most representative deep-sea polynoid subfamily, Macellicephalinae forms a monophyletic group. This subfamily clade comprises two subclades: the Lepidonotopodini clade (BP/PP = 100/1.00), which is specifically restricted to chemosynthetic habitats, and a clade consisting of *Macellicephala*, *Macellicephaloides*, *Gesiella*, etc. Phylogenetic relationships within the latter clade are consistent with the results of genetic distance analysis: *Gesiella jameensis* and Macellicephalinae sp. constitute a sister group, which subsequently forms another sister group with the newly identified species, *Macellicephaloides lingshuiensis* sp. nov. *Macellicephaloides alvini* is phylogenetically distant from *M. lingshuiensis* sp. nov.; instead, it forms a sister group with *Macellicephala* sp. 3 AH 2024, and together they cluster with three other *Macellicephala* species, forming a fully supported clade (BP/PP = 100/1.00).

Polynoinae (BP/PP = 100/1.00): As the most species-rich and morphologically diverse subfamily, Polynoinae is recovered as a monophyletic group with maximum nodal support. It forms a sister relationship with the subfamily Arctonoinae, together comprising a highly supported larger clade (BP/PP = 100/1.00), providing strong evidence for their close evolutionary relationship.

Admetellinae (BP/PP = 100/1.00): Four *Admetella* species form a maximally supported monophyletic clade that further divides into two distinct, well-supported sister lineages, consistent with previous taxonomic work on this genus [39]. The Admetellinae clade is recovered as sister to the subfamily Eulagiscinae with maximum nodal values (BP/PP = 100/1.00).

Lepidonotinae (BP/PP = 100/1.00): As one of the earliest established subfamilies of Polynoidae, Lepidonotinae constitutes a monophyletic clade and is identified as the sister to all the aforementioned clades with strongly supported value (BP/PP = 92/0.99). This phylogenetic relationship corroborated previously phylogenetic work [8,39].

Sigalionidae (BP/PP = 100/1.00): Two selected species of Sigalionidae form a well-supported monophyletic clade, which is recovered as a sister group to the family Polynoidae with high nodal values (BP/PP = 80/0.71). Sigalionidae, together with other outgroups (Acoetidae, Aphroditidae, Eulepethidae, Iphionidae, and Syllidae), strengthen the overall topological support of the phylogeny.

### 3.2. Systematics

Class Polychaeta Grube, 1850
Order Phyllodocida Dales, 1962
Family Polynoidae Kinberg, 1856
Subfamily Macellicephalinae Hartmann-Schröder, 1971

#### 3.2.1. Genus *Macellicephaloides* Uschakov, 1955

Type species. *Macellicephaloides grandicirra* Uschakov, 1955.

Diagnosis. Body relatively small, oval, flattened, with relatively small number of segments (16–21) and 8 pairs of elytra. Elytra smooth, small, not covering dorsum, arranged on segments 2, 4, 5, 7, 9, 1l, 13, and 15.

Prostomium small, bilobed, with 2 palps, and an antenna with ceratophore in middle of prostomium. Lateral antennae and eyes absent. Muscular pharynx with two pairs of subequal dorsal papillae, three pairs of dorsolateral papillae of unequal size with median pair greatly longer than others, and a pair of collar-like ventrolateral folds. Pharynx armed with a fused dorsal jaw consisting of 2 or 3 pieces, and a pair of ventral jaws. First or tentacular segment enclosing prostomium, dorsally with 2 pairs of tentacular cirri, ventrally forming lips of ventral mouth; acicular lobes or chaetae absent. Second segment with uniramous parapodium; ventral cirri inserted basally, distally from lobes of neuropodia. Third segment with a deep mid-ventral depression covered by an oval or rectangular flap. Integument smooth, rarely papillate dorsally, or with middorsal nodular tubercles. Nephridial papillae indistinct. Pygidium enclosed by 2 or 3 posterior segments, with a pair of anal cirri.

Dorsal cirri with moderately to very long, cylindrical cirrophores and short styles. Dorsal tubercles indistinct or rarely nodular. Ventral cirri small, tapering, inserted basally on neuropodia of segment 2; subsequent ventral cirri same size as former, inserted distally on neuropodia. Parapodia elongate, sub-biramous; notopodium reduced as a conical lobe with a more or less projecting, stout aciculum; neuropodium conical with numerous, slender neurochaetae. Neurochaetae numerous, forming fan-shaped bundles. Neurochaetae long, slender, slightly wider basally, distally with two rows of delicate spines forming a shallow furrow.

Remarks. The diagnosis of *Macellicephaloides* was expanded slightly after the work of Pettibone (1976) and (1989) [5,40] to include the species described herein. The elytra can easily become detached or broken due to their delicate and fragile nature, particularly in long-preserved specimens. Due to the complete absence of the elytra, Pettibone only described their number and distribution, without elaborating on their specific morphological characteristics. As some specimens described here retain a few residual elytra, we have incorporated their characteristics into the diagnosis. Pettibone postulated that anal cirri were absent in this genus; however, morphological observations clearly demonstrate their presence in the species described herein.

#### 3.2.2. *Macellicephaloides lingshuiensis* sp. nov. (Figure 2, Figure 3 and Figure 4)

Material examined. *Holotype*. MBM286812, Dive 326, 111°03′16″ E, 17°37′20″ N, 1759 m water depth, 25 September 2024. *Paratypes*. MBM286813, same collection information as above.

Etymology. The specific name is derived from ‘Lingshui’ cold seep, where the species was collected.

Description. Holotype, well-preserved with 18 segments, length 11.35 mm, width 7.57 mm (including chaetae) and 5.27 mm (excluding chaetae). Paratypes with 18 segments, length 7.46–11.11 mm, width 6.27–7.72 mm (including chaetae) and 4.23–4.84 mm (excluding chaetae).

**Figure 2:**
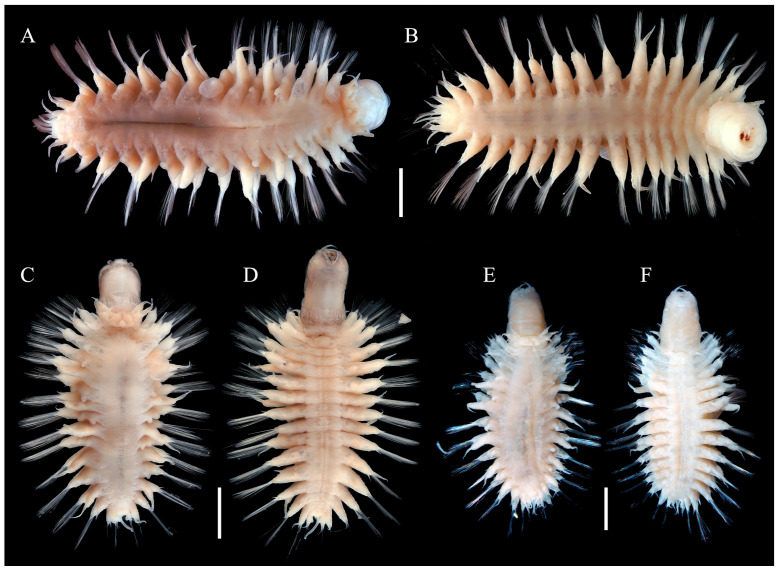
Species of *Macellicephaloides lingshuiensis* sp. nov. in dorsal (**A**,**C**,**E**) and ventral view (**B**,**D**,**F**). (**A**,**B**) holotype, MBM286812; (**C,D**) paratype, MBM286813, specimen 1; (**E**,**F**) paratype, MBM286813, specimen 2. Scale bars: 2 mm (**A**–**F**).

Body (Figure 2) oval shaped, slightly tapering posteriorly; slightly arched dorsally, flattened ventrally. Parapodia (Figure 2) elongated, 2/3 as long as width of body. Elytra (mostly missing) attached to distinct inflated elytrophores (Figure 2A,C,E), 8 pairs, arranged on segments 2, 4, 5, 7, 9, 1l, 13, and 15; elytra small, not covering dorsum, delicate, translucent, surface smooth without tubercles or papillae.

**Figure 3:**
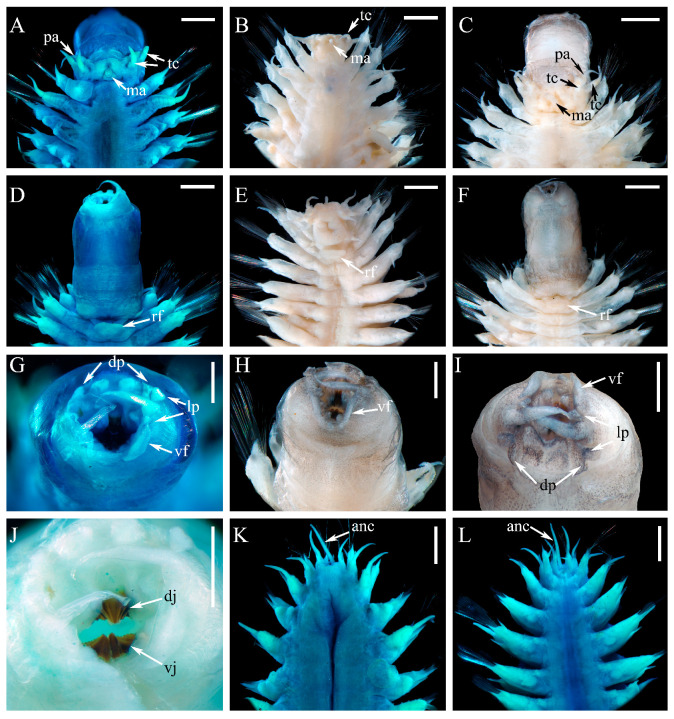
*Macellicephaloides lingshuiensis* sp. nov., holotype (**A**,**D**,**G**,**J**–**L**), paratype specimen 1 (**C**,**F**,**H**,**I**) and paratype specimen 3 (**B**,**E**). (**A**–**C**) Head and anterior segments in dorsal view; (**D**–**F**) Head and anterior segments in ventral view; (**G**) Extended pharynx in anterior view; (**H**) Extended pharynx in ventral view; (**I**) Extended pharynx in dorsal view; (**J**) same as (**G**), showing details of jaws; (**K**) Posterior segments in dorsal view; (**L**) Posterior segments in ventral view. anc: anal cirrus; dj: dorsal jaws; dp: dorsal papillae; lp: lateral papillae; ma: median antenna; pa: palp; rf: rectangular flap; tc: tentacular cirrus; vf: ventrolateral fold; vj: ventral jaws. Scale bars: 1 mm (**A**–**F**,**K**,**L**) and 0.5 mm (**G**–**J**).

**Figure 4:**
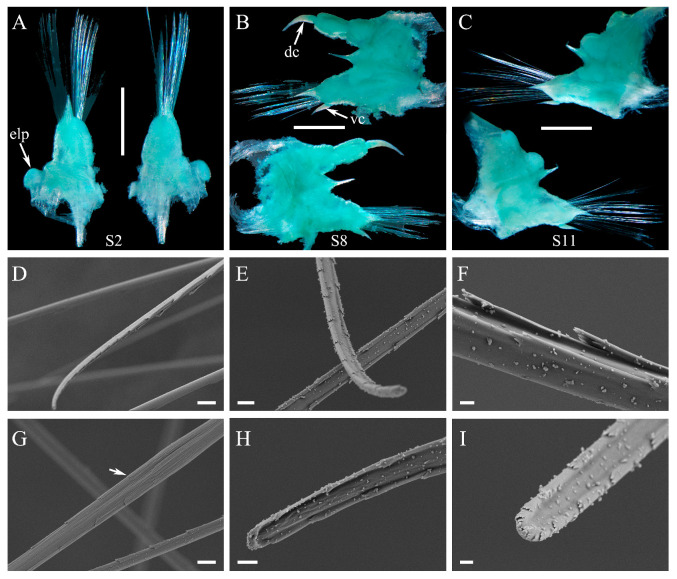
Parapodia and chaetae of *Macellicephaloides lingshuiensis* sp. nov., holotype (**A**–**C**) and paratype specimen 2 (**D**–**I**). (**A**) Right parapodium on segment 2 in posterior (left side) and anterior views (right side), ventral cirrus detached; (**B**) Right parapodium on segment 8 in posterior (lower side) and anterior views (upper side); (**C**) Right parapodium on segment 11 in posterior (lower side) and anterior views (upper side); (**D**) Neurochaetae, showing overall view of the distal part; (**E**,**H**,**I**) Neurochaetae, showing details of the distal part; (**F**) Neurochaetae, showing details of the spines in median part; (**G**) Neurochaetae, arrow showing decreasing of the spines in median part. dc: dorsal cirrus; elp: elytrophores; S2: segment 2; vc: ventral cirrus. Scale bars: 1 mm (**A**–**C**), 10 µm (**D**), 5 µm (**G**), 3 µm (**E**), 2 µm (**H**) and 1 µm (**F**,**I**).

Prostomium (Figure 3A–C) bilobed, much wider than long, withdrawn into tentacular segment. Median antenna (Figure 3A–C) with short and cylindrical ceratophore, inserted in posterior medially of prostomium, with styles short, tapering to slender tips. Lateral antennae absent. Eyes absent. Palps (Figure 3A,C) with large cylindrical palpophores, styles smooth, tapered with fine tips. Pharynx (Figure 3A,C,D,F) robust, with two pairs of dorsal papillae of similar size (Figure 3G,I), three pairs of dorsolateral papillae of unequal size with median pair greatly longer than others (Figure 3G,I), and a pair of collar-like ventrolateral folds (Figure 3G–I); dorsal jaw fused (Figure 3J), with one main large tooth, outer margin serrated; a pair of ventral jaws (Figure 3J), each jaw with one main blunt tooth and outer margin serrated.

First or tentacular segment (Figure 3A–C) fused with prostomium, achaetous, with 2 pairs of tentacular cirri similar in size; tentaculophores short, cylindrical, inserted laterally to prostomium; styles smooth, tapering. Second segment with first pair of elytra (Figure 3A); parapodium (Figure 4A–C) elongate, notopodia absent, neuropodia similar to following ones with bundles of neurochaetae. Third segment with a deep mid-ventral depression covered by a rectangular flap (Figure 3D–F). Integument smooth. Pygidium (Figure 3K,L) with a pair of anal cirri, enclosed by last two parapodia. Nephridial papillae (Figure 3L) indistinct.

Dorsal cirri (Figure 2A,C,E) on cirrigerous segments; cirrophores long, cylindrical; styles smooth, short, tapering. Dorsal tubercles indistinct. Ventral cirri (Figure 3D–F) small, tapering, inserted basally on neuropodia of segment 2; subsequent ventral cirri same size as former, inserted distally on neuropodia.

Parapodia (Figure 4A–C) elongated, flattened, uniramous on segment 2, sub-biramous from segment 3. Notopodia (Figure 4A–C) much shorter than neuropodia, reduced as a conical lobe with a projecting, stout aciculum; notochaetae absent. Neuropodia (Figure 4A–C) elongated, with projecting acicular lobes. Neurochaetae (Figure 4A–C) numerous, forming fan-shaped bundles; Neurochaetae (Figure 4D–I) long, slender, slightly wider basally, blunt distally with two rows of delicate spines forming a shallow furrow.

Type locality. ‘Lingshui’ cold seep of the South China Sea (1759 m depth).

Variation. Some specimens exhibit distinct dark brown stripes (Figure 3C,F,H,I) on the integument of the pharynx, which are absent from the dorsal and ventral surfaces of the body. However, the pharynx of most individuals is unpigmented and lacks color bands (Figure 2A,B,E,F).

All of our specimens are adults. Upon examination, most specimens bear 18 segments. However, the 18th segment is usually small and inconspicuous, making it difficult to detect. In some individuals, either the left or right parapodium of the 18th segment is absent, or both parapodia may be missing. This can lead to misidentification as having 17 segments.

Remarks. Of the nine species described in the genus *Macellicephaloides*, only one, *M. improvisa* Levenstein, 1982, has 18 segments. *Macellicephaloides lingshuiensis* sp. nov. differs from *M. improvisa* in terms of its dorsal cirri and notoaciculae. In the new species, the cirriophores of the dorsal cirri are as long as the styles, whereas in *M. improvisa* they are very short. Furthermore, the notoaciculae are thick and stiff in *M. lingshuiensis*, but thin and flexible in *M. improvisa*.

Most species of *Macellicephaloides* have been found in deep-sea trenches at depths of over 7200 metres. However, only one species, *M. alvini* Pettinbone, 1989 [40], has been found in hydrothermal vents at a depth of approximately 2000 metres. Our new species is the first *Macellicephaloides* species discovered in cold seeps, which represent another chemosynthetic habitat, at a depth of 1759 metres. Morphologically, *M. alvini* is distinguished by up to 16 segments and cirrophores of dorsal cirri bearing pointed projections. In contrast, the new species exhibits 18 segments and smooth cirrophores devoid of projections.

## 4. Discussion

### 4.1. Species Diversity, Distribution Patterns, and Taxonomic Tools of Macellicephaloides

This study describes a new species of *Macellicephaloides* from the Lingshui cold seep in the South China Sea, thus bringing the total number of recognized species in the genus to ten. A review of their biogeographic and bathymetric distributions reveals distinct patterns: all but one species are restricted to the Pacific Ocean, with *M. sandwichensis* Levenstein, 1975 [41] being the sole exception, occurring in the South Sandwich Trench (Atlantic Ocean) at depths of 7200–7934 metres [42]. Within the Pacific Ocean, the distribution of *Macellicephaloides* species further exhibits regional differentiation. Six species are confined to abyssal regions of the western Pacific, inhabiting depths ranging from 7210 to 9950 metres: *M. grandicirra* Uschakov, 1955 [2]; *M. verrucosa* Uschakov, 1955 [2]; *M. vitiazi* Uschakov, 1955 [2]; *M. uschakovi* Levenstein, 1971 [42]; *M. improvisa* Levenstein, 1982 [43]; and *M. villosa* Levenstein, 1982 [44]. In contrast, two species have been documented from the eastern Pacific: *M. alvini* Pettibone, 1989 [40], was discovered in hydrothermal vent habitats of the Gulf of California at a depth of 2004 metres, and *M. moustachu* Bonifácio & Menot, 2018 was identified in the Clarion-Clipperton Fracture Zone (CCFZ) at depths ranging from 4093 to 4978 metres. It is noteworthy that the new species described herein was collected at a depth of 1759 metres, which represents both the shallowest known distribution for *Macellicephaloides* to date (far shallower than the abyssal or deep-sea ranges of its congeners) and the first record of the genus from a cold seep environment (distinct from the hydrothermal vent or abyssal trenches of other species).

The genus *Macellicephaloides* exhibits a remarkable adaptability to deep-sea environments, as evidenced by its occurrence across diverse habitats, including trenches, seamounts, hydrothermal vents, and cold seeps. Furthermore, it has been observed to inhabit a broad bathymetric range spanning from 1759 to 9950 m in the Pacific and Atlantic Oceans [2,6,40,41,42,43]. The ecological and depth versatility exhibited by this genus suggests that its distribution is likely to be far more extensive, potentially encompassing various habitats across the global marine realm. However, our current knowledge remains limited by sampling constraints. Despite the current recognition of ten valid species, the true species diversity of *Macellicephaloides* remains incompletely characterized, highlighting critical gaps in our understanding. This underscores the necessity for sustained deep-sea sampling and integrative research efforts, which are essential for refining estimates of its biodiversity and unraveling the evolutionary drivers behind its ecological success. In order to facilitate future taxonomic investigations and accurate species identification, an updated identification key to all extant species of *Macellicephaloides* is provided herein. This tool serves two objectives: firstly, to facilitate the recognition of extant taxa; and secondly, to encourage the discovery and identification of new species, ultimately enhancing our understanding of this ecologically significant deep-sea genus.

1. Segments 16 or less………………………………………………………………………………………………………………2

Segments more than 16…………………………………………………………………………..4

2. Notopodia with inconspicuous acicular lobes; cirrophores of dorsal cirri bearing pointed projections ………………………………………………………………...*M. moustachu*

Notopodia with prominent, stout acicular lobes; cirrophores of dorsal cirri smooth without projections…………………………………………...……...…...……..……………………..3

3. Dorsal cirri with very long cirrophores and shorter styles, extending as far as or beyond neuropodia; nodular dorsal tubercles on cirrigerous segments 6–14, and middorsal tubercles on segments 3–15.………………………………………………………...*M. verrucosa*

Dorsal cirri with rather short cirrophores and longer styles, not extending beyond neuropodia; nodular dorsal tubercles and middorsal tubercles absent…………………………………………………………....………..............................*M. vitiazi*

4. Segments 17–18……………………………………..……………………….……………...5

Segments 20–21……………………………………………………..…………………………….9

5. Segments 17………………………….……………………………………………………...6

Segments 18.………………………………………………………………………………………8

6. Notoacicula not extra stout or long; pharynx with middle pair of lateral papillae very long; cirrophores of dorsal cirri extending to about tips of neuropodia………………………………………………………………………………..*M. sandwichensis*

Notoacicula long, extending beyond neuropodia in posterior segments; pharynx with middle pair of lateral papillae not extra long……………………………….…………………7

7. Cirrophores of dorsal cirri extending far beyond neuropodia…………..*M. grandicirra*

Cirrophores of dorsal cirri not as long, extending to neuropodia or shorter………..*M. alvini*

8. Notopodia with flexible, thin acicula; cirrophores of dorsal cirri very short…………………………………………………………………………………..*M. improvisa*

Notopodia with stout acicula; cirrophores of dorsal cirri as long as styles…………………………………………………………….………………...*M. lingshuiensis*

9. Dorsal side of body with conical papillae………………………………....…….*M. villosa*

Integument smooth without papillae………………...………...………………….*M. uschakovi*

### 4.2. Morphological and Molecular Distinctions Between Macellicephaloides and Macellicephala

There is no doubt that *Macellicephaloides* and *Macellicephala* are two closely related taxa. Both are early-discovered, deep-sea polynoid genera inhabiting similar abyssal environments. They share morphological characteristics, including a short body, a small number of segments and elytra, an absence of lateral antennae (with only a median antenna remaining), elongated parapodia, and small elytra that can easily be shed. Molecular systematics results further indicate that the two genera are closely related. Recent studies have even shown that *Macellicephaloides* is nested within the *Macellicephala* clade [8]. Despite these morphological and molecular similarities, there are clear morphological differences between the two genera that are sufficient to serve as intergeneric distinctions.

Segments and elytra. Both *Macellicephaloides* and *Macellicephala* are characterized by a reduced number of segments and elytra. *Macellicephala* typically has 18 segments and 9 pairs of elytra, which are distributed across segments 2, 4, 5, 7, 9, 11, 13, 15, and 17. In contrast, the number of segments in *Macellicephaloides* varies from 16 to 21, but there are invariably 8 pairs of elytra distributed across segments 2, 4, 5, 7, 9, 11, 13, and 15. Consequently, all segments from the 16th onward in *Macellicephaloides* species are classified as cirrigerous segments, characterized by the presence of dorsal cirri. Furthermore, there are observable discrepancies in the dimensions of the elytra of *Macellicephaloides* and *Macellicephala*. Despite the fact that the elytra of both genera are easily deciduous, based on the limited number of samples with observable elytra, the elytra of *Macellicephala* are larger and can fully cover the dorsal surface of the body [5], whereas the elytra of *Macellicephaloides* are smaller and unable to cover the dorsal surface (this study).

Pharynx. Polynoids in the subfamily Macellicephalinae usually have a muscular pharynx with two pairs of jaws and a small number of similarly sized papillae. *Macellicephala*, along with other deep-sea genera such as *Bathyeliasona* and *Bathyfauvelia*, is characterized by nine pairs of uniformly sized distal papillae and two pairs of jaws with smooth margins. In contrast, the pharynx of *Macellicephaloides* is highly distinctive, featuring a unique arrangement of papillae (including two pairs of nearly equal dorsal papillae, three pairs of dorsolateral papillae of different sizes with the median pair being significantly longer, and a pair of collar-like ventrolateral folds). It is also armed with a dorsal jaw consisting of two to three fused pieces and a pair of ventral jaws, and both with small teeth on their margins. This unique pharyngeal structure is a key diagnostic feature distinguishing *Macellicephaloides* from other polynoids.

Parapodia and buccal cirri. In both genera, the parapodia are as long as or longer than the width of the body. The notopodia are shorter than the neuropodia, which are elongated with numerous neurochaetae. *Macellicephala* usually has a small number of notochaetae, whereas *Macellicephaloides* has no notochaetae, retaining only a single acicula. Additionally, the buccal cirri of both genera are located at the base of the neuropodia on the second segment. However, buccal cirri of *Macellicephala* are significantly longer than ventral cirri of subsequent segments and resemble the tentacular cirri. By contrast, buccal cirri of *Macellicephaloides* are not elongated and are similar in size to the subsequent ventral cirri.

Nephridial papillae. The nephridial papillae are located on the ventral side, near the base of the neuropodia. These structures are commonly found in deep-sea polynoids, particularly chemosynthetic species in the tribe Lepidonotopodini, and are an important taxonomic feature. The nephridial papillae of *Macellicephala* are first observed on segment 5 and are most prominent on segments 10–12. In contrast, *Macellicephaloides* lacks distinct nephridial papillae on segments 10–12.

These morphological differences, confirmed by our description of *Macellicephaloides lingshuiensis* sp. nov., clearly distinguish *Macellicephaloides* from *Macellicephala*. Significant divergence was also observed at the molecular level between *Macellicephaloides* and *Macellicephala*. The *COI* genetic distance between *Macellicephaloides lingshuiensis* and *Macellicephala* ranged from 18.74% to 22.61%, whereas the intrageneric genetic distance of *Macellicephala* varied from 4.82% to 13.19%. The disparity between the intergeneric and intrageneric genetic distances suggests substantial divergence between the two groups. Phylogenetic analysis further supports this distinction, with *M. lingshuiensis* distinct from the *Macellicephala* clade, thereby substantiating the hypothesis that *Macellicephaloides* and *Macellicephala* represent distinct evolutionary lineages. In contrast, Hiley et al. [8] placed *Macellicephaloides alvini* within the *Macellicephala* clade in their phylogenetic analysis. One possible explanation for this difference is mislabeling (i.e., labelling *Macellicephala* specimens as *M. alvini*), as there was no overlap between the morphological and molecular signatures of *Macellicephaloides* and *Macellicephala* in our dataset. However, without access to the voucher specimens from Hiley et al.’s study, this remains a speculative interpretation; re-examination of the vouchers would be necessary to verify the specimens’ taxonomic identity and resolve this inconsistency.

### 4.3. Systematics and Phylogeny of Macellicephalinae and Related Groups

Deep-sea polynoids, exhibiting rich morphological diversity, were categorized into numerous subfamilies and genera by early taxonomists based on morphological traits [45,46]. The subfamily Macellicephalinae is the earliest established and the most diverse among deep-sea polynoid subfamilies [7]. Despite extensive phylogenetic analyses of deep-sea polynoids by various researchers [6,8,29,33,47,48,49], the systematics of Macellicephalinae and related taxa have undergone frequent revisions, with key controversies persisting to date.

In an analysis incorporating morphological and molecular data, Bonifácio & Menot [6] synonymized 10 subfamilies lacking lateral antennae within Macellicephalinae, namely Bathyedithinae, Bathymacellinae, Branchinotogluminae, Branchiplicatinae, Branchipolynoinae, Lepidonotopodinae, Macellicephaloidinae, Macelloidinae, Polaruschakovinae, and Vampiropolynoinae. However, subsequent research by Hatch et al. [50] challenged this broad synonymy, emphasizing the monophyly of the deep-sea polynoids inhabiting chemosynthetic habitats and reinstated the subfamily Lepidonotopodinae, which encompasses most chemosynthetically associated polynoids. The aforementioned classification system incorporates the previously recognized Branchinotogluminae, Branchiplicatinae, Branchipolynoinae, and Lepidonotopodinae, in addition to *Bathykurila* and *Levensteiniella*—genera that were initially assigned to Macellicephalinae. Building on these debates, Gonzalez [49] conducted a transcriptome-based phylogenetic analysis, which revealed that Lepidonotopodinae sensu Hatch et al. [50] is nested within Macellicephalinae. This finding was corroborated by Hiley et al. [8], who once again synonymized Lepidonotopodinae with Macellicephalinae and reclassified it as the tribe Lepidonotopodini within Macellicephalinae.

Although Hiley et al. [8] clarified the taxonomic status of polynoids from chemosynthetic habitats, the phylogenetic relationships of other taxa within the subfamily Macellicephalinae remain ambiguous. The present study corroborated that Lepidonotopodinae sensu Hatch et al. [50] is nested within the Macellicephalinae clade. Furthermore, it elucidated the evolutionary relationships between *Macellicephaloides* and other related taxa. We suggest that *Macellicephaloides* and *Macellicephala* represent two distinct evolutionary lineages, supported by morphological comparisons and *COI* gene analyses. *Macellicephaloides* is more closely related to the genus *Gesiella*, which is endemic to anchialine caves. Due to its adaptation to a swimming lifestyle, *Gesiella* has distinctly different prostomium and parapodia (e.g., lateral antennae located on the ventral side of the prostomium; elongation of dorsal cirri). Early taxonomists considered it to be similar to the subfamily Polynoinae, which are commonly found in coastal waters. They even established a separate subfamily for it (Gesiellinae Muir, 1982) [51]. The genus *Pelagomacellicephala*, which has also been observed in anchialine caves, represents the sister to the clade composed of *Macellicephala*, *Macellicephaloides* and *Gesiella*. This phylogenetic placement further indicates a close evolutionary relationship between deep-sea polynoids and their cave-dwelling counterparts.

Since our phylogenetic tree includes only two species of *Macellicephaloides*, one of which may be mislabeled, we are unable to assess the monophyly of *Macellicephaloides*. However, based on the morphological phylogenetic analysis of deep-sea polynoids conducted by Bonifácio & Menot [6], we hypothesize that *Macellicephaloides* is a monophyletic group. Future phylogenetic studies will require more extensive sampling of species (particularly of key deep-sea genera like *Macellicephala* and *Macellicephaloides*) and datasets to clarify the phylogenetic relationships within Macellicephalinae. Our study, while providing a robust phylogenetic framework, highlights persistent uncertainties in the deeper nodes of the subfamily’s phylogeny. Nevertheless, the current work—and the phylogeny to be published in the future—will contribute significantly to understanding the evolutionary history, morphological character evolution, and true diversity of this ecologically important deep-sea group.

## Data Availability

The original contributions presented in this study are included in the article/Supplementary Material. Further inquiries can be directed to the corresponding author.

## Supplementary Materials

The following supporting information can be downloaded at: https://www.mdpi.com/article/10.3390/cimb47110897/s1.
